# Rethinking the electrographical boundaries of status epilepticus: controversies and paradoxes of the ictal-interictal continuum

**DOI:** 10.3389/fneur.2026.1802653

**Published:** 2026-03-27

**Authors:** Pilar Bosque-Varela, Nicolas Jannone-Pedro, Lukas Machegger, Eugen Trinka, Panagiota-Eleni Tsalouchidou

**Affiliations:** 1 Department of Neurology, Christian Doppler University Hospital, Centre for Cognitive Neuroscience, European Reference Network EpiCARE, Paracelsus Medical University of Salzburg, Salzburg, Austria; 2 Centre for Cognitive Neuroscience, Neuroscience Institute, Christian Doppler University Hospital, Paracelsus Medical University of Salzburg, Salzburg, Austria; 3 Department of Clinical Neurophysiology, University and Polytechnic La Fe Hospital, European Reference Network EpiCARE, Valencia, Spain; 4 Section of Neuropediatrics, University and Polytechnic La Fe Hospital, European Reference Network EpiCARE, Valencia, Spain; 5 Department of Neuroradiology, Christian Doppler University Hospital, Paracelsus Medical University, Salzburg, Austria; 6 Karl Landsteiner Institute for Clinical Neurosciences, Salzburg, Austria; 7 Second Department of Neurology, School of Medicine, “Attikon” University Hospital, National and Kapodistrian University of Athens, Athens, Greece; 8 Epilepsy Center Hessen, Faculty of Medicine, Department of Neurology, Philipps-Universität Marburg, Marburg, Germany

**Keywords:** critical care EEG, non-convulsive status epilepticus, peri-ictal MRI abnormalities, peri-ictal neuroimaging abnormalities, seizures

## Abstract

The ictal–interictal continuum (IIC) challenges the traditional dichotomous classification of electroencephalographic activity into ictal and interictal states and represents a major zone of diagnostic and therapeutic uncertainty in status epilepticus (SE). IIC is defined by rhythmic and periodic electroencephalographic (EEG) patterns that do not fulfill formal seizure criteria and occupies a gray zone in which the interpretation of what is ictal, treatment responsiveness, and risk of neuronal injury remains controversial. In this narrative review, we explored the boundaries between SE and the IIC, focusing on key controversies and paradoxes that emerge across electroclinical scenarios and neuroimaging findings. More specifically, we examine the time-locked electroclinical correlates and antiseizure medication responsiveness as markers of ictality, ongoing controversies in EEG-based definitions, and the role of peri-ictal neuroimaging abnormalities as complementary markers of metabolic burden. This review aims to summarize these topics and discuss key gaps for future research in the management of IIC.

## 1 Introduction

Ictal–interictal continuum (IIC) is a purely electroencephalographic (EEG) definition, most recently updated by the American Clinical Neurophysiology Society’s (ACNS) Standardized Critical Care EEG terminology (1). According to ACNS criteria, three electrographic patterns are classified within the IIC when lasting ≥10 s and not qualifying as definite epileptic seizures or definite status epilepticus: (1) periodic discharges (PDs) or spike-and-wave/sharp-and-wave (SW) patterns occurring at frequencies >1.0–2.5 Hz; (2) PDs or SW patterns at frequencies of 0.5–1.0 Hz when accompanied by a plus modifier or demonstrable fluctuation; and (3) lateralized rhythmic delta activity (LRDA) averaging >1 Hz with a plus modifier or fluctuation. In the ACNS Standardized Critical Care EEG Terminology, “plus modifiers” refer to additional features used to further characterize rhythmic and periodic patterns (RPPs) by indicating superimposed features such as fast activity (+F), rhythmic delta activity (+R), or sharp waves (+S).

This definition has contributed to a standardized communication among clinicians and conceptually represents a grey zone between interictal and ictal patterns. However, distinguishing ictal from interictal remains crucial to start early intervention and prevent irreversible neuronal injury beyond the critical time point T2 of status epilepticus (SE) (2–4).

These patterns pose substantial diagnostic and therapeutic challenges in daily clinical practice, with many unresolved questions, including which EEG patterns on the IIC warrant treatment, how aggressively they should be treated, whether they are due to potential reversible etiologies such as metabolic disturbances or drug intoxication (e.g., cefepime or metaldehyde) (5, 6), and whether these patterns represent harmful ictal activity or epiphenomena of underlying brain injury (7, 8). The absence of definitive biomarkers and evidence-based treatment strategies, mainly driven by the lack of clinical trials, further complicates decision-making (8).

Beyond these uncertainties, the IIC is also characterized by several conceptual controversies and paradoxes across different domains, including clinical presentation, electroencephalographic patterns and neuroimaging features, which may ultimately contribute to a deeper understanding of this spectrum (8–12). These topics are addressed in this narrative review.

## 2 Clinical presentations and treatment definitions

### 2.1 The paradox of electroclinical time-locked correlate

A definite clinical correlate that is time-locked to an EEG pattern defines an electroclinical seizure; when the duration criterion is met, this is regarded as electroclinical status epilepticus (SE), while by definition, IIC lacks time-locked clinical manifestations (1, 13, 14). This finding highlights the value of clinical–EEG concordance as a pragmatic marker of ictal activity. At the same time, interpretation of this criterion benefits from consideration of an important factor: the anatomical localization of ictal activity, which strongly influences whether a clinical correlate can be detected (15).

When ictal activity involves eloquent cortical regions, including primary motor, sensory, language, or visual areas, they are more likely to generate overt, time-locked clinical manifestations, such as clonic motor activity and are thus more readily recognized as ictal (15). Conversely, ictal activity arising from clinically “silent” regions, such as prefrontal or frontopolar structures, may generate no or only very subtle clinical manifestations (16, 17). Consequently, the perceived “ictality” of EEG patterns that do not fulfill formal seizure criteria, such as the IIC, depends strongly on anatomical localization and its associated clinical symptoms, rather than necessarily on fundamental differences in underlying pathophysiology. However, localization-based electroclinical definitions are insufficient to determine the metabolic burden or risk of neuronal injury, indicating that SE categorization in these cases does not reliably capture the potential for brain damage.

Similarly, clinical response to IIC patterns, which is often used to support classification as SE (18), may itself be influenced by the anatomical localization of ictal activity. When the activity involves non-eloquent cortical regions, resolution or attenuation of EEG abnormalities may not be accompanied by clear clinical improvement, as the ictal activity may not have been responsible for overt neurological signs in the first place (19). In such cases, absence of a clinical response may be misinterpreted as evidence against “ictality,” rather than reflecting limitations of clinical assessment in detecting seizures arising from clinically silent regions (9). In critically ill patients, this challenge is further compounded by reduced quantitative consciousness (somnolence, sopor, coma), sedative medications, neuromuscular blocking agents, mechanical ventilation, and baseline neurological deficits produced by the etiology per se, which may mask subtle manifestations. As a result, the absence of a time-locked clinical sign should be interpreted cautiously, particularly when EEG patterns arise from regions with limited or inaccessible clinical expression (9, 19).

### 2.2 The paradox of electroclinical response definitions in the ASM/BZD trial

In both ACNS critical care EEG terminology and the Salzburg Consensus Criteria (SCC) (1, 13, 14), improvement after a parenteral benzodiazepine (BZD) or antiseizure medication (ASM) is used as supportive evidence for “ictality,” particularly when electrographic seizure or electroclinical seizure criteria are not met. More specifically, IIC patterns are considered nonconvulsive SE (NCSE) when a concomitant EEG and clinical response is observed, whereas the absence of these falls in the category of possible NCSE or IIC (18, 20). In contrast, in definite SE, failure to respond to ASM/BZD is interpreted as treatment refractoriness, rather than “non-ictality” (9, 21). This divergence implies that the interpretation of treatment response depends on the initial electroclinical diagnosis, creating a paradox that is largely driven by how the underlying etiology is weighted in explaining the clinical presentation in IIC and possible NCSE (9). This is particularly relevant in critically ill patients, in whom impaired consciousness, sedative exposure, and underlying structural or metabolic injury may obscure both EEG and clinical responsiveness, thereby further limiting the reliability of treatment response as a discriminator between ictal and non-ictal states (19).

## 3 EEG-based definitions in IIC

### 3.1 Controversies in EEG boundaries between electrographic SE and IIC

The distinction between electrographic SE and IIC rests on how electrographic seizures are defined by the current ACNS Standardized Critical Care EEG Terminology, based on Salzburg Consensus Criteria (SCC): (1) any evolving pattern, or (2) epileptiform discharges at >2.5 Hz (1, 13, 14). These criteria were validated in cohorts that included a substantial proportion of patients with pre-existing epilepsy (38% in the validation group and 52% in the control group) (22), which likely contributes to their good performance in that population. For example, the >2.5 Hz cutoff for non-evolving discharges is well-suited for the diagnosis of typical absence SE (around 3 Hz), which occurs in patients with idiopathic generalized epilepsy (23).

However, a prior history of epilepsy is reported in only 3 to 23% of critically ill patients with RPPs (24–26), which may help explain why the 2.5 Hz threshold may exclude patients in this setting (21). For instance, lateralized periodic discharges (LPDs) at low frequencies (≤1.5 Hz) are more common than those at higher frequencies, yet they are still associated with both a high probability of seizures and secondary neuronal damage (25, 27, 28). In addition, the same criterion also excludes epileptic encephalopathies presenting with atypical absence SE (1.5 to 2.5 Hz) (29). Although speculative, one potential explanation is that acute or chronic brain injury from the underlying etiology may limit the cortex’s ability to generate higher-frequency activity, raising the possibility that some lower-frequency patterns in this context may still represent SE (30). Nevertheless, broadening electrographic SE definitions to include low- or intermediate-frequency discharges would increase the likelihood of incorporating dysfunctional patterns such as triphasic waves (31).

### 3.2 Controversies in IIC “ictality” based on EEG: increased risk of seizures

As previously noted, the definition of IIC encompasses specific combinations of RPPs, frequency thresholds, and the presence of “plus” modifiers or fluctuation (1). Importantly, IIC is not merely a third category between interictal and ictal patterns; it is a spectrum in which some patterns are “more ictal” than others (32). “Ictality” based on EEG is commonly operationalized as the risk of subsequent clinical seizures (i.e., ictogenesis) during continuous EEG, and the same variables that define inclusion within the IIC framework largely drive this risk. Specifically, higher frequencies (>2 Hz), lateralized RPPs (especially LPDs) and “plus” modifiers are well-established predictors (28), that have been integrated into the 2HELPS2B score (33).

**Figure 1:**
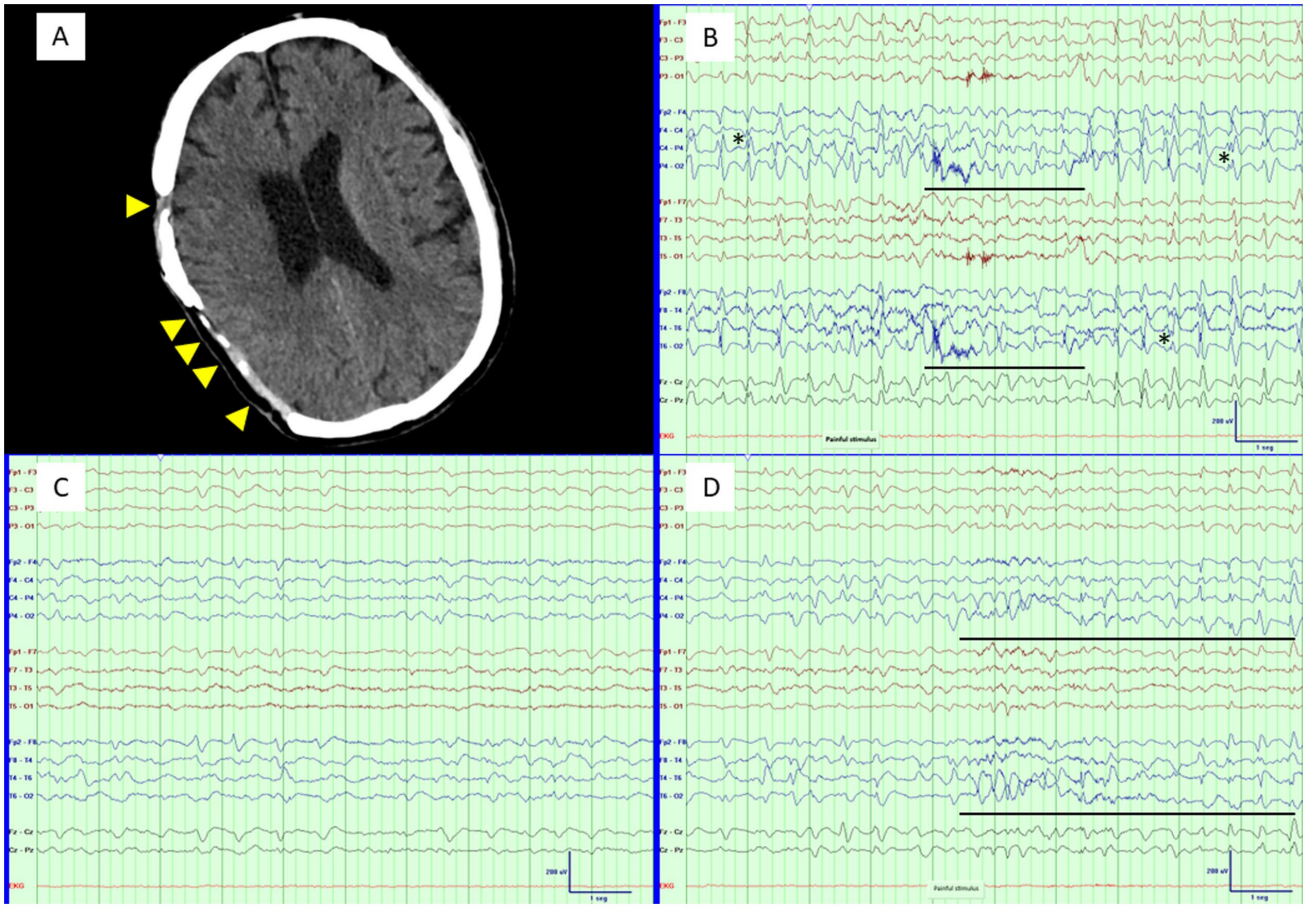
Stimulus-induced IIC pattern. **(A)** Prominent parietotemporal skull defect (arrowheads). **(B)** LPDs with triphasic morphology at 1.8 Hz, maximal in the right posterior quadrant (IIC). ‘False’ lateralization and superimposed fast activity (*) are attributable to a prominent breach effect; therefore, the pattern is best interpreted as equivalent to GPDs (also IIC). Stimulation produces transient theta activity that replaces the main pattern (stimulus-terminated; underlined). **(C)** A BZD is administered, resulting in an EEG response (only a few brief trains of LPDs) without clinical improvement (consistent with sleep induction). **(D)** Stimulation is repeated, revealing transient theta activity followed by increased discharge amplitude and spikiness (stimulus-induced; underlined). Because the pattern was consistent with typical triphasic waves (‘falsely’ lateralized by a breach effect), toxic–metabolic encephalopathy was suspected; hyperammonemic encephalopathy was confirmed (serum ammonia, 179 μmol/L). A 1 Hz low frequency filter, 70 Hz high frequency filter, 50 Hz notch filter, and 10 μV/mm sensitivity were applied to the EEGs.

Nevertheless, the term “lateralized” denotes activity that originates from one side, but which may manifest as bilateral asymmetry due to the volume conduction effect (1). If the cause of lateralization is another one, the original source of the pattern should be considered in EEG interpretation (34). One illustrative example is generalized periodic discharges (GPDs) that manifest as LPDs due to a breach effect (Figure 1). Furthermore, the association between seizures and plus modifiers has not been researched for each modifier separately, except for LPDs+F (35, 36); thus, it remains unclear whether certain modifiers convey a higher risk than others. Moreover, fluctuation reflects pattern dynamics that approach evolution and were incorporated into IIC criteria as equivalent to plus modifiers, but its risk for epileptic seizures has not been demonstrated yet (1, 14). Similarly, sharply contoured LPDs may carry a higher risk of seizures than blunt LPDs, that warrants further consideration (35).

**Figure 2:**
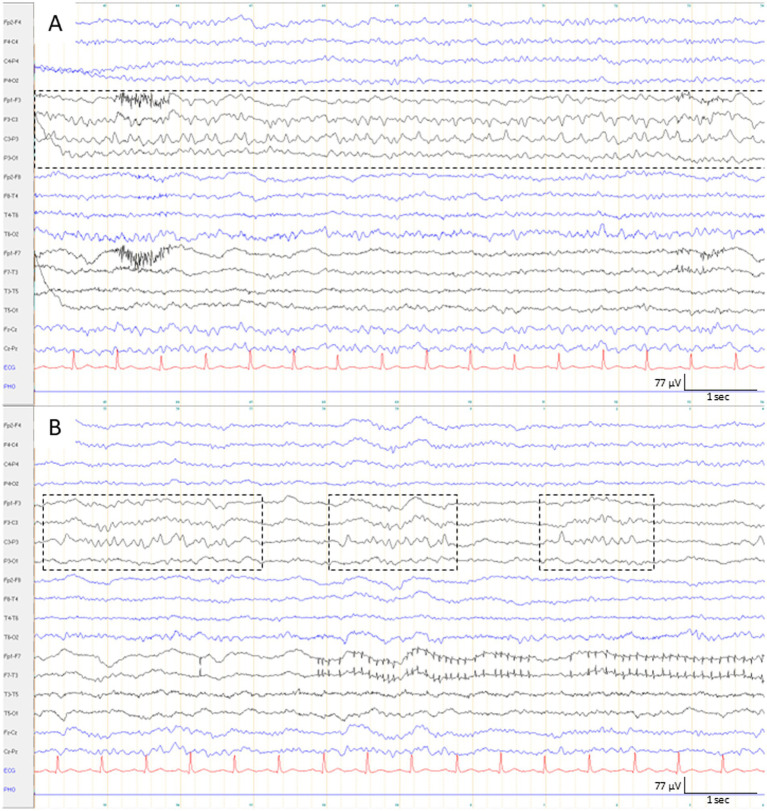
Seizure and BIRDs. **(A)** Ten-second EEG epoch demonstrating the initial part of an epileptic seizure (duration ≥10 s, dashed box). The seizure is characterized by evolving rhythmic theta activity over the left central region. **(B)** The same recording demonstrates BIRDs lasting approximately 1–3 s, with localization and morphology very similar to the seizure shown in panel **(A)** (dashed boxes). These bursts are not classified as seizures because they do not meet the ≥10-s duration criterion. EEGs were displayed using a 1 Hz low-frequency filter, 70 Hz high-frequency filter, 50 Hz notch filter, and a sensitivity of 7 μV/mm.

Brief potentially ictal rhythmic discharges (BIRDs) are rhythmic bursts faster than delta activity lasting <10 s; they should be evolving, similar in location or morphology to interictal epileptiform discharges, or sharply contoured (1, 37). BIRDs carry the highest seizure-risk weight in the 2HELPS2B score (33) and would be classified as seizures if a clinical correlate were present (1, 37). Therefore, they can be viewed as electrographic “very brief seizures” that fall short of the arbitrary ≥10 s duration criterion for seizures (Figure 2). Future work should evaluate whether a modest reduction in the duration threshold could allow some BIRDs to be reclassified as electrographic seizures while still excluding interictal epileptiform discharges from the seizure definition.

### 3.3 Controversies in IIC “ictality” based on EEG: decreased risk of seizures

EEG features that have been associated with lower ictogenesis include triphasic morphology and stimulus-induced changes. In several studies, GPDs with triphasic morphology were linked to a lower seizure risk than non-triphasic GPDs (31, 38), although other work did not replicate this difference (39). These mixed findings may reflect variability in “triphasic morphology” definition, underscoring the need for further refinements of this term (40). One possibility is to place greater emphasis on the key defining feature—highest amplitude of the second (positive) phase—rather than the number of phases, which can vary from two to four (1, 31, 41). Notably, stimulus-related changes occur in approximately half of cases exhibiting this morphology (31). Indeed, triphasic morphology, induction due to stimuli, and generalized distribution (GPDs) are the core characteristics of so-called “typical triphasic waves,” which have been proposed to be “less ictal” than those with atypical features (Figure 1) (34, 42). Therefore, incorporating the concept of “triphasic waves” into IIC definitions may enable a more refined assessment of “ictality” across the spectrum.

Other RPPs may also be stimulus-induced and still meet IIC criteria. This phenomenon is not a modality-specific evoked response but rather the appearance or exacerbation of an EEG pattern following arousal, which may be triggered by diverse stimuli or occur spontaneously (1). Such patterns are termed stimulus-induced rhythmic, periodic and ictal-appearing discharges (SIRPIDs) and were initially interpreted as a marker of “cortical hyperexcitability” (43). However, the association with seizures appears to depend on factors other than stimulus-induced changes per se. Specifically, SIRPIDs have been linked to SE only when they constitute a lateralized or evolving pattern (43). Similarly, for each RPP considered separately—generalized rhythmic delta activity (GRDA), LRDA, GPDs, and LPDs—the presence or absence of stimulation-induced changes did not affect seizure risk (28). Furthermore, SIRPIDs tend to manifest after a NCSE or clinical seizures and after successful treatment of those events (43, 44). Accordingly, an alternative interpretation is that they represent a “suppressed epileptic phenomenon” (i.e., less ictal), which only emerges after arousals (44). Consistent with this view, SIRPIDs have not demonstrated hyperperfusion on SPECT in available studies (45). Collectively, these observations support the idea that stimulus-related changes may serve as a marker of “low ictality” across the IIC—an interpretation that has already been suggested specifically for triphasic waves (34).

Encephalopathic patterns such as typical triphasic waves and GRDA have been considered non-ictal, because of their comparatively lower seizure risk (28, 31, 34). Nonetheless, these patterns sometimes display more ictal characteristics. For example, atypical triphasic waves may constitute a pattern of SE (5, 42), and GRDA may sometimes be associated with a clinical correlate and considered ictal (1, 46). In addition, computational models suggest that common mechanisms may generate both encephalopathic patterns and generalized NCSE, supporting the conceptual inclusion of triphasic waves and GRDA within the IIC (47, 48). From this perspective, “typical” encephalopathic patterns would reside toward the more interictal part of the spectrum, where BZD/ASM treatment is unlikely to confer clinical benefit. By contrast, patterns at the more ictal end may worsen the altered mental state —so-called “boundary syndrome”—in which treatment may be warranted (4, 42).

## 4 Neuroimaging in IIC

### 4.1 Can we resolve the electroclinical dilemmas with neuroimaging?

Over recent years, neuroimaging has become a fundamental pillar in the management of SE. It supports diagnostic assessment when EEG findings are equivocal, particularly within the IIC (49, 50), contributes to prognostic evaluation (51, 52), and assists in delineating associated brain injury (2, 3). In addition, neuroimaging findings have been increasingly explored as potential predictors of subsequent epilepsy development (52). Despite this expanding role, substantial uncertainties persist regarding the interpretation of imaging abnormalities and their clinical implications, underscoring the need for cautious and contextualized interpretation.

Peri-ictal neuroimaging abnormalities (PNA) represent the main imaging correlates of SE and can be detected across multiple modalities, including structural MRI as well as functional techniques such as Positron Emission Tomography (PET), Single-Photon Emission Computed Tomography (SPECT), and CT perfusion (CTP). Large prospective studies have reported PNA in up to 50% of patients with definite SE (53). Among these, the most frequently reported finding is regional hyperperfusion, most commonly demonstrated using arterial spin labeling (ASL) (53, 54). According to the literature, ASL hyperperfusion is observed in approximately 89% of patients with definite NCSE (49). Other commonly described MRI abnormalities include diffusion-restricted lesions and cortical or subcortical hyperintensities on FLAIR sequences, which occur in up to 51 and 36% of patients, respectively, according to our previous meta-analysis (49).

Functional imaging tools have likewise demonstrated hyperperfusion or hypermetabolism in a substantial proportion of patients (11, 55, 56). These findings are generally interpreted as indirect markers of ongoing epileptic activity and are thought to reflect early stages of the underlying pathophysiological cascade associated with sustained neuronal firing and metabolic stress (57).

Nevertheless, neuroimaging cannot be regarded as a gold standard for the diagnosis of SE. Clear electrographic SE may occur in the absence of detectable imaging abnormalities in approximately 50% of cases, even when neuroimaging is performed within the first 48 h after SE onset (53). Conversely, PNA may be observed in patients with equivocal or non-classical EEG patterns, including those classified within the IIC (58).

Importantly, a recent study comparing PNA in patients with definite SE and those with IIC (59) found no statistically significant differences between the two groups. These findings suggest a substantial overlap in imaging features and raise the unresolved question of whether SE and IIC represent distinct entities or rather different expressions along a shared pathophysiological spectrum. Supporting this notion, a recent PET study in a heterogeneous cohort of patients with diverse etiologies, including both definite SE and IIC with various EEG patterns, demonstrated hypermetabolism without a clear association with the frequency of epileptiform discharges (58). The authors concluded that discharge frequency alone may not adequately capture the true metabolic burden of epileptic activity. Indeed, some patients of this cohort did not even fulfill the criterion for IIC and had mostly interictal EEGs.

**Figure 3:**
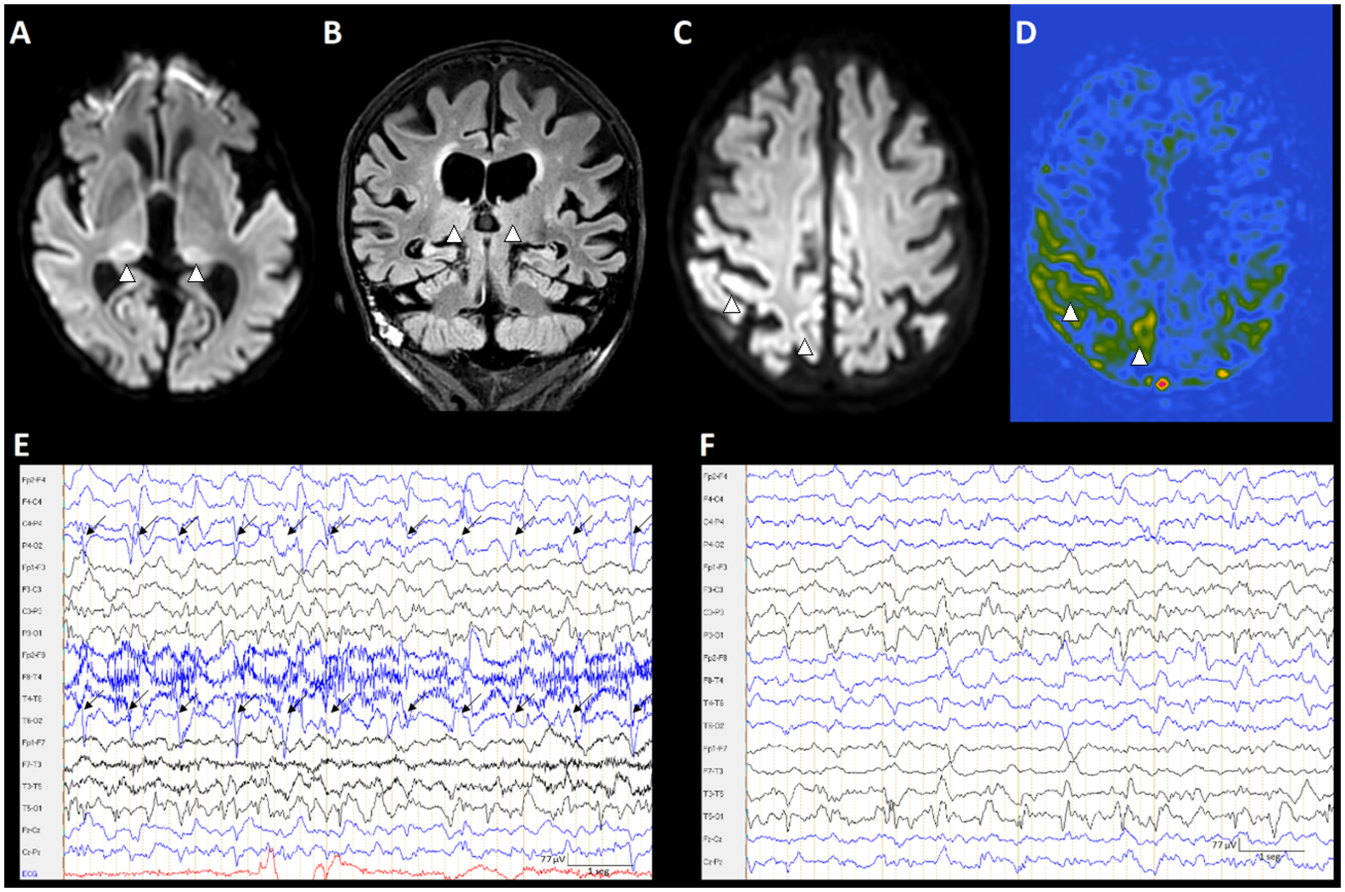
Patient with a focal to bilateral tonic–clonic seizure lasting 3 min, followed by decreased level of consciousness progressing to coma. MRI demonstrated typical PMA, and EEG findings were consistent with IIC. Bilateral pulvinar involvement with concordant ASL hyperperfusion and associated EEG changes. Top row: MRI **(A–D)**. Bottom row: EEG **(E,F)**. **(A)** Axial diffusion-weighted imaging (DWI) demonstrates bilateral diffusion restriction in the pulvinar nuclei (arrowheads). **(B)** Coronal FLAIR shows bilateral pulvinar hyperintensity; due to slight asymmetry of the coronal slice angulation, the right pulvinar appears mildly brighter than the left (arrowheads). **(C)** Axial DWI reveals laminar cortical diffusion restriction in the right parietal cortex (arrowheads). **(D)** Arterial spin labeling (ASL) shows focal hyperperfusion spatially concordant with the diffusion-restricted cortex in **(C)** (arrowheads). **(E)** EEG demonstrates lateralized periodic discharges with superimposed fast activity (LPDs + F; 1.1 Hz) over the right posterior quadrant (arrows). **(F)** EEG after treatment. EEGs were displayed using a 1 Hz low-frequency filter, 70 Hz high-frequency filter, 50 Hz notch filter, and a sensitivity of 7 μV/mm.

Consistent with these observations, LPDs, a core feature of the IIC, have been strongly associated with hyperperfusion (54, 59), hypermetabolism (58), and PNA (59, 60). Although LPDs are also related to a high risk of seizures in continuous EEG (28, 33), the association between other seizure predictors, such as discharge frequency with cerebral hypermetabolism, remains unclear, with earlier studies suggesting a positive association (61) and more recent work finding none (58, 59). This paradoxical dissociation between seizure propensity and metabolic burden supports a model of partially independent pathophysiological axes and suggests that discharge frequency alone may not fully capture the metabolic burden of epileptiform activity (7). In the Salzburg cohort of patients with IIC, although not statistically significant, there was a tendency of higher hyperperfusion in patients with LPDs in lower frequencies (59) (Figure 3).

### 4.2 Imaging mimics of peri-ictal neuroimaging abnormalities

PNA can be used with promising results to support the diagnosis of SE (59). Although PNA may overlap with etiological imaging features, PNA can be diagnosed in most cases by recognizing typical patterns, including non–vascular territory distribution, highly specific locations such as the pulvinar or hippocampus (2), and concomitant hyperperfusion (62). Furthermore, in diffusion-restricted lesions, quantitative assessment of DWI and ADC signal intensity ratios—calculated by comparing the lesion signal with the contralateral normal-appearing brain tissue—may further aid differentiation. In this context, DWI ratios <1.495 and ADC ratios >0.735 support the diagnosis of peri-ictal MRI abnormalities (PMA) (63).

**Figure 4:**
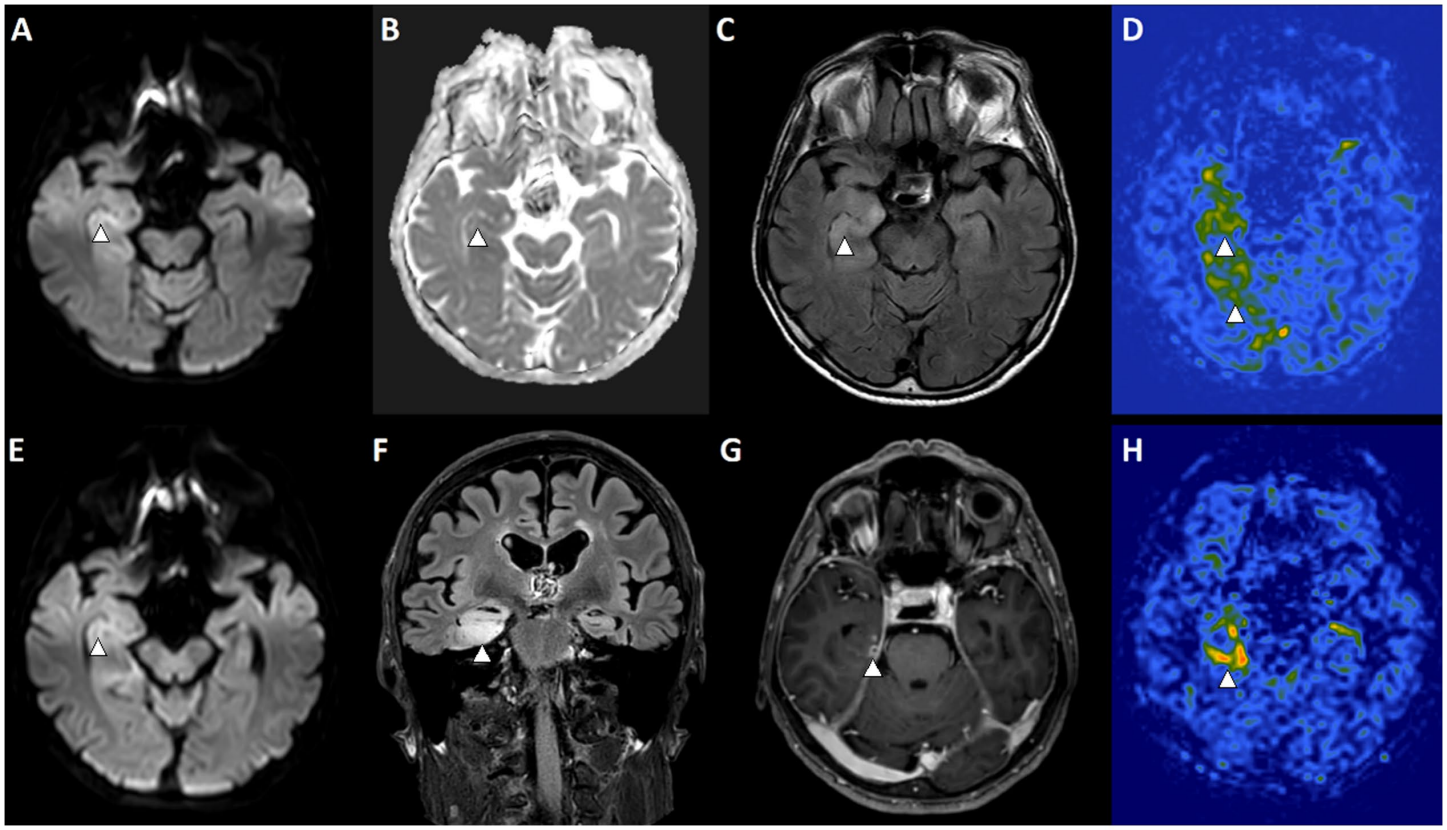
Overlap of PMA-like imaging features and encephalitis-related MRI findings in a patient with status epilepticus. Baseline MRI **(A–D)** and 7-day follow-up MRI **(E–H)** are shown. **(A)** Axial diffusion-weighted imaging (DWI) demonstrates a subtle diffusion abnormality in the right hippocampus/mesial temporal region (arrowhead). **(B)** The corresponding apparent diffusion coefficient (ADC) map shows only minimal signal reduction, consistent with a mild diffusion restriction pattern (arrowhead). **(C)** Axial FLAIR reveals right mesial temporal hyperintensity involving the hippocampus and uncus, compatible with edema/inflammation (arrowhead). **(D)** Arterial spin labeling (ASL) shows concordant hyperperfusion in the right hippocampal/parahippocampal region, consistent with PMA-like perfusion changes (arrowheads). **(E)** Follow-up DWI after 7 days demonstrates persistent subtle diffusion abnormality in the right hippocampus (arrowhead). **(F)** Coronal FLAIR shows marked progression of right mesial temporal edema (arrowhead). **(G)** Post-contrast T1-weighted imaging demonstrates new enhancement in the right mesial temporal region, supporting an encephalitis pattern (arrowhead). **(H)** Follow-up ASL demonstrates spatially congruent hyperperfusion relative to the FLAIR abnormality, consistent with persistent PMA-like perfusion features (arrowhead).

Nonetheless, most episodes of SE are associated with a structural or functional brain abnormality, including acute primary insults to the central nervous system (CNS), remote symptomatic lesions, or progressive neurological disorders, with truly cryptogenic SE representing only a minority of cases (64). This presents two key challenges in interpreting neuroimaging: first, differentiating abnormalities attributable to the underlying etiology from those directly induced by SE, and second, determining the relative contribution of PNA versus the primary pathological process (Figure 4).

Several conditions can closely mimic PNA. Subacute ischemic stroke, for instance, may show transient “luxury hyperperfusion” on perfusion MRI or SPECT (65). In addition, stroke-related abnormalities often involve restricted diffusion on DWI during the acute phase and T2/FLAIR hyperintensity in the subacute phase, which may overlap with peri-ictal cortical changes (63). Interpretation becomes even more challenging when the EEG shows an IIC pattern that could be driven by the underlying stroke lesion rather than ongoing seizure activity.

Another example is the acute encephalitis, both autoimmune (66) and viral, can produce cortical and limbic T2/FLAIR and DWI abnormalities, contrast enhancement, and regional hyperperfusion of the hippocampus (67). Among affected regions, the hippocampus has been reported as the second most frequently involved in PMA and appears highly specific for SE-related changes (2). This highlights a substantial overlap between etiological and SE-related imaging findings, emphasizing that the underlying cause can simultaneously explain both the observed MRI abnormalities and the occurrence of SE (Figure 3 displays a case of acute encephalitis and SE showing overlapping of PMA and encephalitis changes).

Brain neoplasms represent another important mimic of PNA. Cortical and peritumoral T2/FLAIR hyperintensities and focal hyperperfusion (68) can be observed in regions adjacent to high-grade gliomas or metastases, reflecting a combination of vasogenic peritumoral edema and tumor-related ictal activity. These imaging findings may transiently overlap with PNA, particularly when subtle or nonconvulsive seizure-like symptoms occur in close temporal association with tumor progression, surgical intervention, or treatment-related changes.

Finally, mitochondrial encephalopathy, lactic acidosis, and stroke-like episodes (MELAS) syndrome as well as other mitochondriopathies (69) provide a particularly illustrative example. Stroke-lesions in MELAS often present with migratory cortical and subcortical hyperintensities, may be reversible over days to weeks, sometimes leading to focal brain atrophies, and frequently localize to regions similar to those affected by PMA, fulfilling the imaging and temporal dynamics typically described for SE patients. Given that individuals with MELAS often experience recurrent or prolonged and refractory episodes of SE, in particular NCSE, it is challenging to distinguish PMA from MELAS lesions. Although speculative, this could indeed represent more extensive PMA, blurring the line between metabolic disorders and PNA (70).

Future studies should include control groups with similar underlying pathologies but without SE, using multimodal imaging to better isolate peri-ictal neuroimaging abnormalities attributable specifically to SE. Longitudinal designs integrating advanced imaging with continuous EEG will be essential to clarify the temporal dynamics, reversibility, and clinical relevance of these changes.

## 5 Summary of key controversies and future directions

The IIC remains a diagnostic and therapeutic challenge because of its fluid boundaries with SE. As discussed throughout this review, several conceptual paradoxes arise when clinical presentation, treatment response, EEG criteria, and neuroimaging findings are considered together.

From a clinical perspective, the absence of time-locked manifestations in IIC may not necessarily exclude ictal activity, particularly when epileptic discharges arise from clinically silent cortical regions or when neurological examination is limited in critically ill patients. Likewise, response to benzodiazepines or antiseizure medications may support ictality, whereas lack of response may reflect treatment refractoriness or confounding factors rather than non-ictal activity.

Electroencephalographically, the main controversy concerns the thresholds separating electrographic SE from IIC. Although current criteria provide a clear standardized framework, certain patterns, particularly lower-frequency periodic discharges, may still be associated with seizure risk or metabolic burden. These observations support the concept that IIC represents a spectrum in which some patterns appear to be more ictal than others.

Although neuroimaging is not part of the current diagnostic definitions of SE or IIC, imaging findings add an important dimension by demonstrating that some IIC patterns may be associated with substantial metabolic demand and PNA thereby providing complementary information to electroclinical assessment.

These uncertainties are further compounded by the complex and dynamic nature of rhythmic and periodic EEG patterns, the heterogeneity of underlying brain etiologies, and limited evidence regarding optimal treatment strategies. Future prospective studies integrating continuous EEG, advanced neuroimaging, and clinical outcomes will be essential to define ictality better, optimize diagnostic thresholds, and guide treatment decisions.

## 6 Conclusion

Current EEG criteria for NCSE and IIC provide an essential framework for management, but remain challenging to apply across heterogeneous clinical contexts, including the critical care setting. Rather than relying on a single modality such as EEG, clinical presentation, response to ASM trial, or neuroimaging features, a multimodal approach integrating all of these is likely to offer the most comprehensive understanding of these conditions and may support more accurate diagnosis and guide management of IIC. Future research in this direction is warranted.
